# Pilot Quasi-Randomized Controlled Study of Herbal Medicine Hochuekkito as an Adjunct to Conventional Treatment for Progressed Pulmonary *Mycobacterium avium* Complex Disease

**DOI:** 10.1371/journal.pone.0104411

**Published:** 2014-08-05

**Authors:** Yasunori Enomoto, Eri Hagiwara, Shigeru Komatsu, Ryuichi Nishihira, Tomohisa Baba, Hideya Kitamura, Akimasa Sekine, Atsuhito Nakazawa, Takashi Ogura

**Affiliations:** Department of Respiratory Medicine, Kanagawa Cardiovascular and Respiratory Center, Yokohama, Japan; Glaxo Smith Kline, Denmark

## Abstract

**Introduction:**

Hochuekkito, a traditional herbal medicine, is occasionally prescribed in Japan to treat patients with a poor general condition. We aimed to examine whether this medicine was beneficial and tolerable for patients with progressed pulmonary *Mycobacterium avium* complex (MAC) disease.

**Methods:**

This pilot open-label quasi-randomized controlled trial enrolled 18 patients with progressed pulmonary MAC disease who had initiated antimycobacterial treatment over one year ago but were persistently culture-positive or intolerant. All patients continued their baseline treatment regimens with (n = 9) or without (n = 9) oral Hochuekkito for 24 weeks.

**Results:**

Baseline characteristics were generally similar between the groups. Most patients were elderly (median age 70 years), female, had a low body mass index (<20 kg/m^2^), and a long-term disease duration (median approximately 8 years). After the 24-week treatment period, no patient achieved sputum conversion. Although the number of colonies in sputum tended to increase in the control group, it generally remained stable in the Hochuekkito group. Radiological disease control was frequently observed in the Hochuekkito group than the control group (8/9 vs. 3/9; p = 0.05). Patients in the Hochuekkito group tended to experience increase in body weight and serum albumin level compared with those in the control group (median body weight change: +0.4 kg vs. −0.8 kg; median albumin change: +0.2 g/dl vs. ±0.0 g/dl). No severe adverse events occurred.

**Conclusions:**

Hochuekkito could be an effective, feasible adjunct to conventional therapy for patients with progressed pulmonary MAC disease. Future study is needed to explore this possibility.

**Trial Registration:**

UMIN Clinical Trials Registry UMIN000009920

## Introduction

*Mycobacterium avium* complex (MAC) is the most common pathogen involved in pulmonary nontuberculous mycobacterium diseases. Recommended treatment regimens offer combinations of drugs including macrolides and others. Patients should ideally be treated for a long period until confirmation of at least 1 year of negative sputum cultures [1]. Majority of patients on macrolide-containing regimens are able to achieve negative sputum cultures within 12 months [2], [3]. However, some cases are intractable despite appropriate therapy [4] or due to an intolerance of the recommended therapeutic agents. For those cases, few options are left other than symptom palliation. Although surgery is one of those [1], [5], it is difficult to perform it in patients with advanced disease. Alternative and feasible therapeutic agents are awaited.

Hochuekkito (Bu-Zhong-Yi-Qi-Tang in Chinese) is a herbal mixture used in Kampo traditional medicine; it is composed of several plant materials, including *Angelicae radix*, *Astragali radix*, *Atractylodis rhizoma*, *Aurantii nobilis pericarpium*, *Bupleuri radix*, *Cimicifugae rhizoma*, *Ginseng radix*, *Glycyrrhizae radix*, *Zingiberis rhizoma*, and *Zizyphi fructus*. This drug is often used in Japan to treat patients who are in a weakened physical condition, although its pharmacological activity and mechanism of action are not completely understood. Previous studies have demonstrated the immunomodulating effects by increasing serum interferon-γ level [6], [7], which is advantageous for killing intracellular pathogens like MAC. On the other hand, Hochuekkito can potentially improve nutritional status and quality of life [8], [9]. Actually, beneficial effects of this medicine were reported on some respiratory diseases including chronic lower respiratory tract infection [10] and chronic obstructive pulmonary disease [8]. Therapeutic strategy against chronic wasting disease by immunomodulating activities and/or improving nutritional status would be of a future for progressed MAC disease even without bactericidal effect. In this regard, we conducted a pilot comparative study to evaluate the efficacy and safety of Hochuekkito as an adjunct to conventional treatment in those patients with progressed intractable pulmonary MAC disease.

## Methods

### Patients

This study was conducted in accordance with good clinical practice and the ethical principles outlined in the Declaration of Helsinki. The protocol was approved by the institutional review board of Kanagawa Cardiovascular and Respiratory Center. All patients provided their written informed consent before registration. The trial was registered under number UMIN000009920 (http://www.umin.ac.jp/). The protocol for this trial and supporting CONSORT checklist are available as supporting information; see Checklist S1 and Protocol S1.

Eligible patients were ≥20 years of age and had been diagnosed with pulmonary MAC disease in accordance with American Thoracic Society/Infectious Diseases Society of America criteria [1] at least 1 year prior to the time of registration. The patients had been treated with antimycobacterial drugs for ≥1 year at our institution but were persistently culture-positive or intolerant and thus met the recommendations for Hochuekkito therapy. Kampo drugs are generally recommended to be used on the basis of the patient's “Sho,” which is the special concept in this field and implies the symptoms and/or systemic conditions. Clinicians assess the “Sho,” such as Yang (positivity)/Yin (negativity) or Jitsu (fullness)/Kyo (deficiency), and thereafter, choice the most appropriate drug. The recommendation for Hochuekkito therapy included a decline in physical strength, general fatigue, or appetite loss, which are also originated from the concept of “Sho.” MAC-positive sputum cultures in solid media within the 3 months prior to registration were confirmed in all patients. Drug susceptibility tests were performed with the broth microdilution method. Macrolides, rifamycins, ethambutol, aminoglycosides, and fluoroquinolones were all regarded as antimycobacterial drugs in this study.

Exclusion criteria included ongoing malignant diseases, interstitial lung diseases or other severe pulmonary diseases, human immunodeficiency virus infection, pregnancy or childbearing potential, and cases in which the antimycobacterial regimen had been changed or who had initiated therapy with herbal drugs (including Hochuekkito) within the 3 months before registration.

### Study design and treatment

This was an open-label, single-center, prospective, parallel group, comparative study. The eligible patients were enrolled by each clinician, and sequentially allocated to Hochuekkito or control groups in order at the secretariat of our institution. Patients were followed at our institution every 4–8 weeks for up to 24 weeks after allocation. Allocation was stratified by whether patients were currently receiving antimycobacterial medications at the time of registration. All patients continued their preceding treatment regimens at the same dosages over the follow-up period. Those in the Hochuekkito group also received oral Hochuekkito 5.0 g twice daily or 7.5 g three times daily before or after each meal. Daily dosage was determined by each clinician on the basis of patients' physical constitution and/or demands. If a patient had not been prescribed any antimycobacterial drugs at the time of registration, he or she was observed with no medication (control group) or was medicated with Hochuekkito alone (Hochuekkito group).

### Efficacy endpoints

The primary endpoint was the sputum conversion rate at 24 weeks after treatment initiation. Sputum culture conversion was defined as confirmation of culture negativity at two consecutive time points (on different days but within a 2-week period). If the patients could not produce sputum, inducement by nebulized hypertonic salt solution was performed. All sputum cultures were grown on solid media, which enables counting the number of colonies. The microbiology technicians were unaware of the treatment assignments throughout the study. The number of MAC colonies in the sputum cultures was also recorded before and after treatment. Because of difficulty counting the exact number of colonies, we used a semi-quantitative scoring system: 0 (no colonies), 1 (1–9 colonies), 2 (10–49 colonies), 3 (50–99 colonies), 4 (100–199 colonies), 5 (200–299 colonies), 6 (300–399 colonies), 7 (400–499 colonies), and 8 (≥500 colonies). Important secondary endpoint was changes in chest X-ray images over the 24-week treatment period. Chest X-ray images were independently reviewed by two experienced clinicians (S. K. and R. N.) who were blinded to the group assignment; they evaluated the disease extent (<1/3, 1/3–2/3, or >2/3 of the lung area) and the presence/absence of cavities at baseline as well as changes over the 24-week treatment period (improved, stable, or worsened). Disagreements between the two reviewers were resolved by consensus. Additional and exploratory secondary endpoints were changes in chronic obstructive pulmonary disease assessment test (CAT: 0–40) score [11] evaluating patients' symptom, body weight, and three biomarkers over the 24 weeks: serum albumin, C-reactive protein (CRP), and erythrocyte segmentation rate (ESR). In the field of pulmonary MAC disease, evidenced symptom scoring system is not available. Although the validation was not studied, we used CAT score for the substitution.

### Safety assessment

Safety was assessed by recording adverse events at least every 8 weeks. These were obtained by clinicians' interview and laboratory results including complete blood counts and the following serum biomarkers: aspartate aminotransferase, alanine aminotransferase, total bilirubin, direct bilirubin, urea nitrogen, creatinine, sodium, and potassium. The severity of each event was determined by the Common Terminology Criteria for Adverse Events v4.0 (CTCAEv4).

### Statistical analysis

We assumed negative conversion rates for 5% in the control group and 30% in the Hochuekkito group. With a two-sided alpha level of 0.05, total enrollment of 78 patients was needed to attain power of 80%. Thus, the target sample size was determined as 80. Efficacy and safety were assessed among all allocated patients. Data were expressed as number of patients or as medians with observed ranges. Interobserver agreement with respect to the radiological assessment was determined using kappa statistics (<0.4: relatively poor; 0.4–0.6: moderate; 0.6–0.8: good; 0.8–1.0: excellent). Group comparisons were performed using Fisher's exact test, Pearson's test, or Mann-Whitney U test as appropriate. Serial changes in the number of colonies in sputum culture were evaluated by Wilcoxon signed-rank test. A p value of <0.05 was considered to be significant. Analyses were performed using SPSS software version 13.0 (SPSS, Inc., Chicago, IL, USA).

## Results

### Baseline characteristics

**Figure 1:**
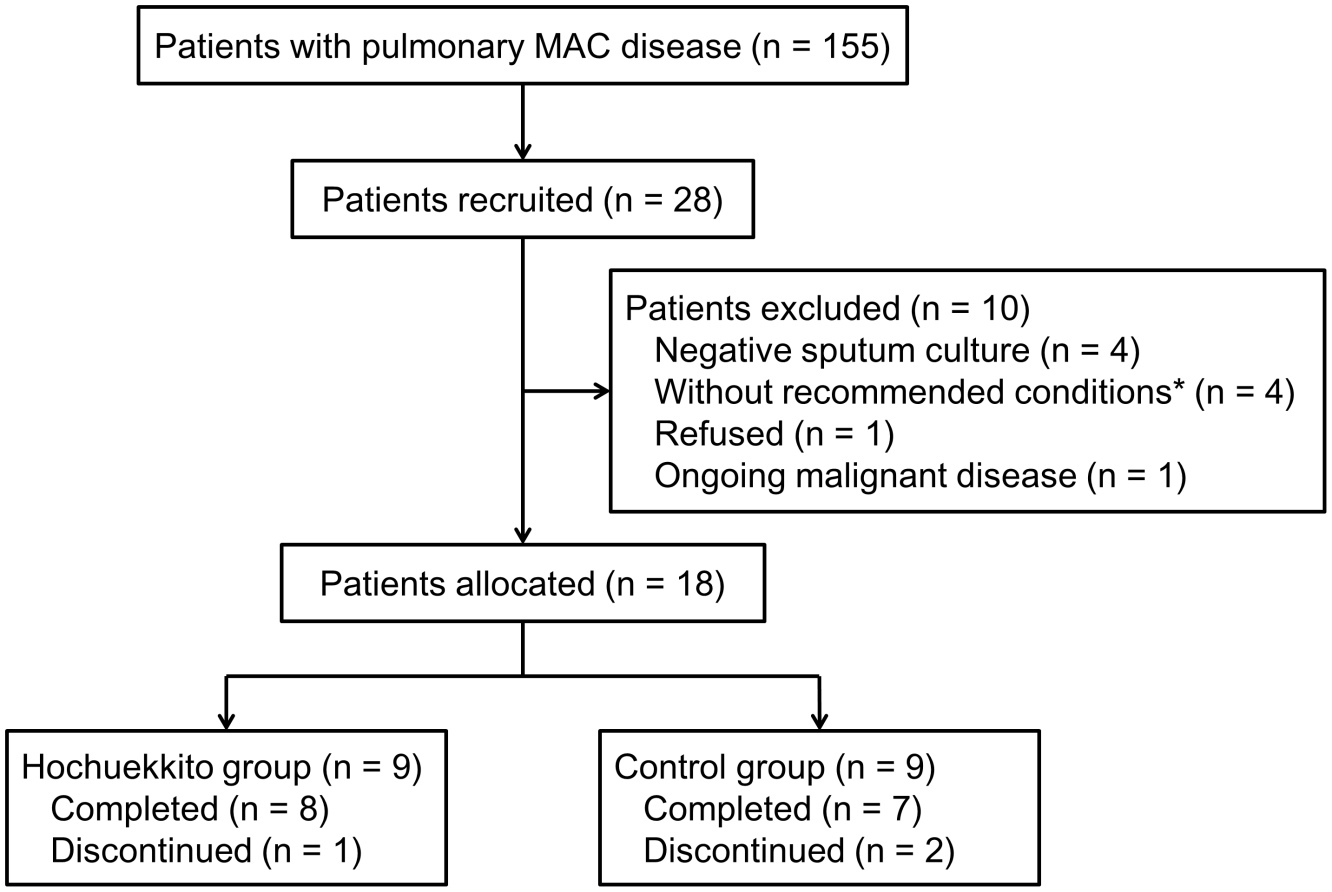
Patient inclusion/exclusion. MAC  =  *Mycobacterium avium* complex. *Declined physical strength, general fatigue, or appetite loss.

Of the 155 patients with pulmonary MAC disease who were diagnosed and followed up at our institution, 28 were recruited for candidates between February and June 2013. Eighteen of these patients were eligible for inclusion and were allocated to the Hochuekkito group (n = 9) or the control group (n = 9) (Figure 1). We were unable to recruit as many patients as expected, and thus decided to end the study with smaller number of patients than the protocol planned. Baseline characteristics are summarized in Table 1, and are mostly similar between the two groups. Most patients were elderly (median age 70 years), female, had a low body mass index <20 kg/m^2^, and a long-term disease duration (median approximately 8 years). There were no significant differences in chest X-ray findings between the two groups. Interobserver agreement with respect to the radiological assessment was moderate to excellent (disease extent, κ = 0.45; presence/absence of cavities, κ = 1.0). The baseline ESR was significantly higher and the serum albumin level was comparatively lower, in the Hochuekkito group compared with the control group. There were no significant differences in the number of MAC colonies in the sputum cultures and baseline CAT scores between the groups.

**Table 1:** Comparison of baseline characteristics in the Hochuekkito and control groups.

	Hochuekkito (n = 9)	Control (n = 9)	p value
Age, years	70 (44, 80)	70 (59, 83)	0.60
Sex, male / female	2/7	2/7	1.00
Smoking status, current and former / never	1/8	4/5	0.29
BMI, kg/m^2^	17.6 (16.8, 25.7)	17.4 (15.0, 23.7)	0.40
Disease duration, months	96 (18, 241)	90 (28, 128)	1.00
Number of colonies in sputum culture*	4 (1, 8)	4 (1, 8)	0.50
Extent in chest X-ray, <1/3/1/3–2/3/>2/3	2/2/5	1/2/6	0.81
Cavity in chest X-ray, yes / no	7/2	4/5	0.34
Albumin, g/dl	3.8 (3.5, 4.1)	4.2 (3.6, 4.5)	0.12
CRP, mg/dl	0.62 (0.05, 6.49)	0.19 (0.01, 1.38)	0.20
ESR, mm/hr	60 (9, 100)	35 (11, 49)	0.047
CAT score†	11 (3, 27)	17 (4, 30)	0.43

Data are expressed as the number of patients or median (with observed range: minimum, maximum). All p values are for comparisons between the two groups and were obtained by Fisher's exact test, Pearson's test, or Mann-Whitney U test as appropriate.

BMI  =  body mass index; CRP  =  C-reactive protein; ESR  =  erythrocyte segmentation rate; CAT  =  chronic obstructive pulmonary disease assessment test.

*Semi-quantitative scoring system: 0 (no colonies), 1 (1–9 colonies), 2 (10–49 colonies), 3 (50–99 colonies), 4 (100–199 colonies), 5 (200–299 colonies), 6 (300–399 colonies), 7 (400–499 colonies), and 8 (≥500 colonies).

† Chronic obstructive pulmonary disease assessment test score ranging from 0 to 40. A higher score means more severe symptoms [11].

Treatment status at the time of registration is summarized in Table 2. Each group included one patient who had not received any antimycobacterial medication at the time of registration. A three-drug regimen consisting of rifampicin, ethambutol, and clarithromycin was most common in both groups. Aminoglycosides and/or fluoroquinolones were frequently added to the combination regimen in the Hochuekkito group. However, there was no significant difference in the number of drugs patients had been administered before study initiation [median 4 (range 3–10) in the Hochuekkito group vs. median 4 (range 1–5) in the control group]. Most patients had experienced aminoglycosides administration in past. Drug sensitivity tests identified 8 clarithromycin-resistant MAC cases (more than 32 µg/ml minimum inhibitory concentration) among the entire cohort of patients; these were evenly distributed between the two groups.

**Table 2:** Comparison of treatment status at the time of registration between patients in the Hochuekkito and control groups.

	Hochuekkito (n = 9)	Control (n = 9)
On medication, yes / no	8/1	8/1
Baseline treatment regimens		
RFP, EB, CAM + others*	3	0
RFP, EB, CAM	3	5
RFP, CAM, LVFX	1	1
RFP, CAM	1	1
EB, CAM	0	1
CAM resistant†, yes / no	4/5	4/5

Data are expressed as number of patients.

RFP  =  rifampicin (10 mg/kg/day); EB  =  ethambutol (15 mg/kg/day); CAM  =  clarithromycin (15–20 mg/kg/day); LVFX  =  levofloxacin (10 mg/kg/day).

*Aminoglycosides (Kanamycin or Streptomycin: 15 mg/kg two/three times per a week) and/or fluoroquinolones.

† Minimum inhibitory concentration to CAM >32 µg/ml by the broth microdilution method.

### Efficacy

**Figure 2:**
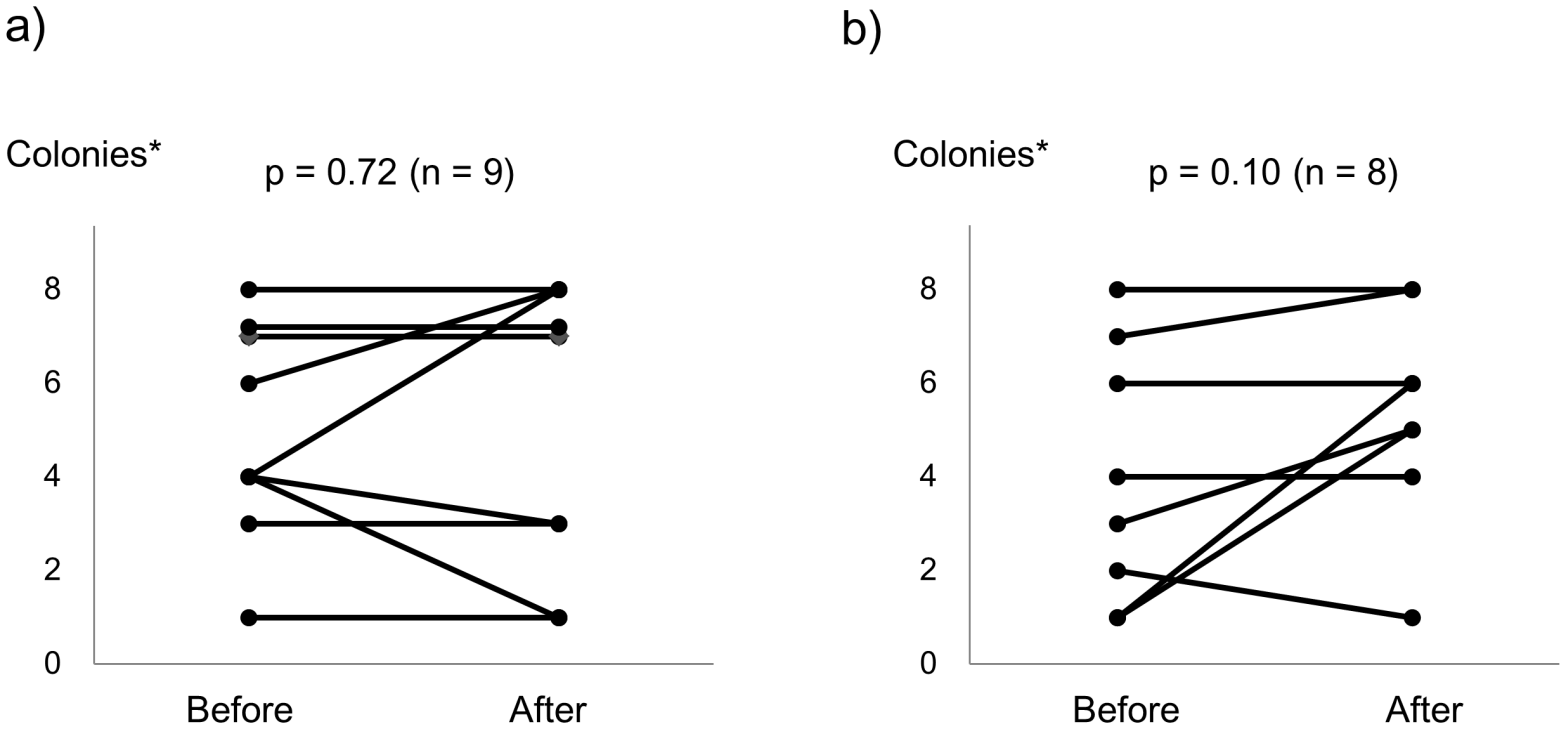
Changes in sputum findings over the 24-week treatment period. The number of MAC colonies in the sputum cultures of patients in the Hochuekkito (a) and control (b) groups. All p values were determined by using Wilcoxon signed-rank test. MAC  =  *Mycobacterium avium* complex. *Semi-quantitative scoring system: 0 (no colonies), 1 (1–9 colonies), 2 (10–49 colonies), 3 (50–99 colonies), 4 (100–199 colonies), 5 (200–299 colonies), 6 (300–399 colonies), 7 (400–499 colonies), and 8 (≥500 colonies).

After the 24-week treatment period, no patients in either group achieved sputum conversion (95% confidence interval: 0.0–33.6%). The post-treatment sputum of one patient in the control group could not be assessed because of contamination. Changes in the number of colonies in sputum culture are shown in Figure 2. There were no statistically significant changes in either group, although the numbers of colonies were relatively increased in the control group but tended to remain stable in the Hochuekkito group.

**Figure 3:**
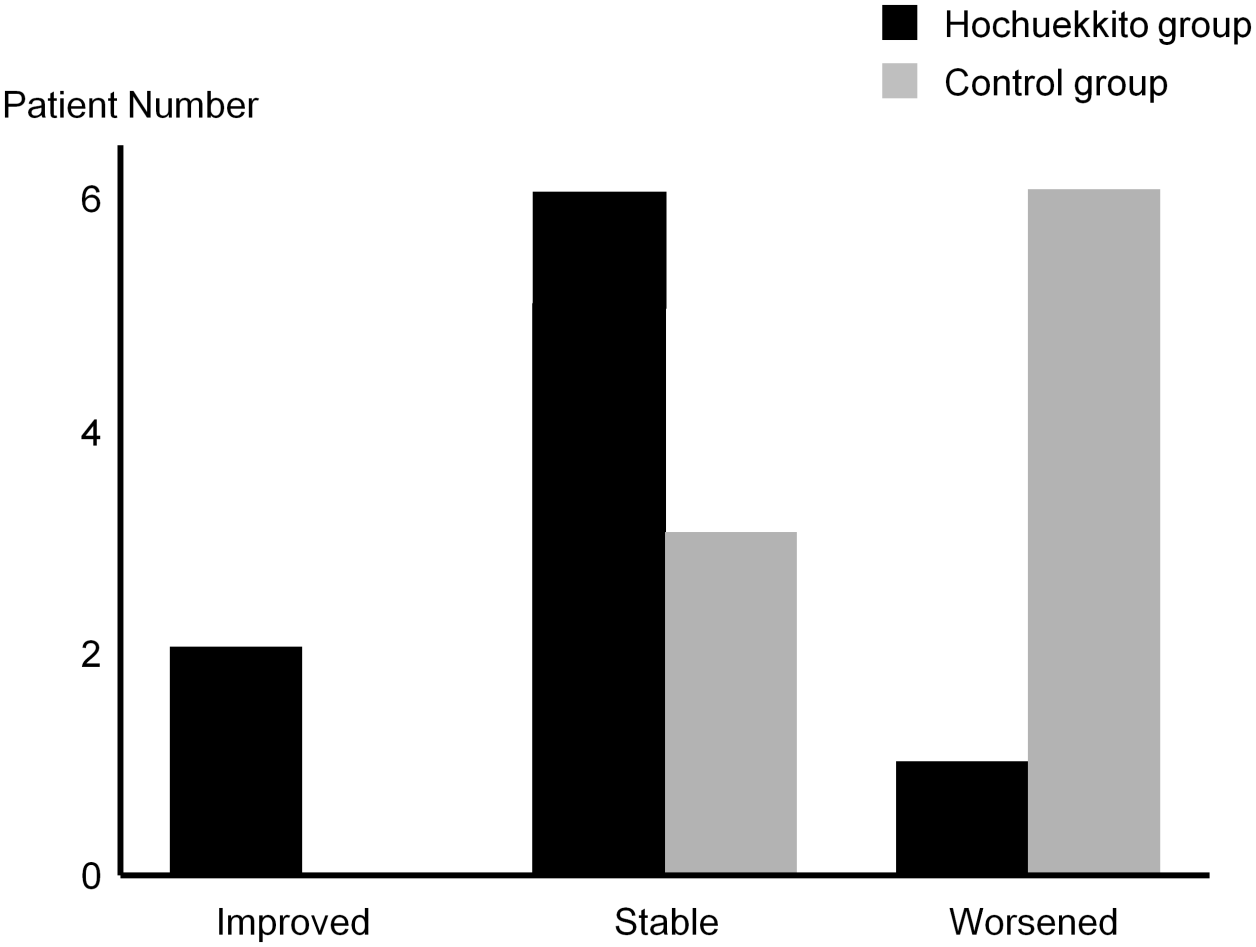
Changes in radiological findings over the 24-week treatment period. Radiological assessment was performed by comparing chest X-ray images before and after treatment.

**Figure 4:**
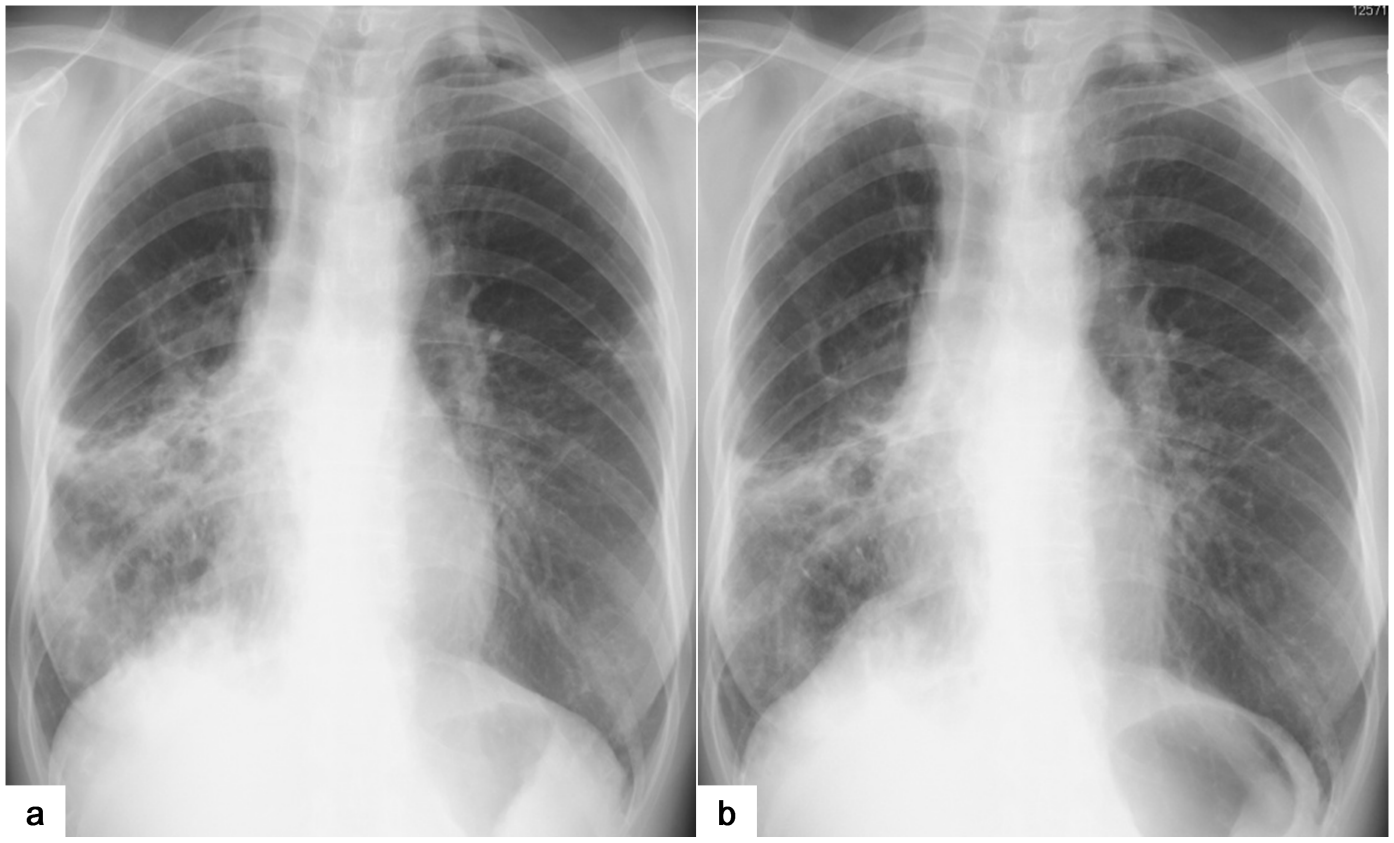
Chest X-ray images of a 61-year-old woman in the Hochuekkito group who achieved radiological improvement. The images shown are before (a) and after 24 weeks of (b) treatment. The consolidation areas in the bilateral lower lesions were decreased after treatment.

There was good interobserver agreement regarding changes in chest X-ray images (κ = 0.72). Most patients in the Hochuekkito group were categorized as “improved” or “stable” by the radiological assessment, but those in the control group were more frequently categorized as having a “worsened” condition (Figure 3). Disease control (according to the radiological assessment) was greater in the Hochuekkito group compared with the control group (8/9 vs. 3/9; p = 0.05). The chest X-ray images of a patient in the Hochuekkito group who was categorized as “improved” is shown in Figure 4. Changes in other secondary and exploratory endpoints over the 24 weeks are summarized in Table 3. CAT score, CRP, and ESR were increased or worsened in most patients of both groups despite the 24-weeks treatment. On the other hand, changes in body weight and serum albumin level seemed to be different. That is, patients in the Hochuekkito group tended to experience increase in body weight and serum albumin level compared with those in the control group (median body weight change: +0.4 kg vs. −0.8 kg; median albumin change: +0.2 g/dl vs. ±0.0 g/dl). Interestingly, patients who achieved improvement in X-ray image all gained weight, whereas most of the patients whose X-ray image was worsened lost weight in both groups.

**Table 3:** Comparison of secondary endpoints between the Hochuekkito and control groups over the 24-week treatment period.

Group/No.	Age/Sex	X-ray	CAT*	BW, kg	ALB, g/dl	CRP, mg/dl	ESR, mm/hr
Hochuekkito							
1	61/F	Improved	+8	+0.9	+0.2	+0.49	+15
2	80/F	Stable	−1	+0.4	−0.5	−0.02	−17
3	44/M	Worsened	+5	−0.7	−0.2	−1.53	+24
4	78/F	Stable	−5	−0.5	+0.3	−0.01	−18
5	63/M	Stable	+3	+2.0	+0.4	+0.20	+9
6	77/F	Stable	+5	±0.0	+0.5	+0.06	+6
7	54/F	Stable	+11	−3.8	−0.5	−0.13	+30
8	70/F	Stable	+3	+1.0	+0.3	+0.71	+18
9	73/F	Improved	−2	+1.4	+0.2	+0.19	+7
Median			+3	+0.4	+0.2	+0.06	+9
Control							
1	73/F	Worsened	+4	±0.0	−0.2	+0.01	+5
2	70/F	Stable	+9	−0.8	±0.0	+0.91	+55
3	70/F	Worsened	−3	+0.8	+0.1	±0.00	+22
4	69/M	Worsened	+7	−2.9	−0.3	+2.37	+44
5	83/M	Worsened	+1	±0.0	−0.5	+4.73	+52
6	67/F	Stable	−1	+0.9	±0.0	−0.01	+1
7	81/F	Worsened	−6	−−2.3	−0.2	−0.15	+10
8	59/F	Stable	+4	−1.5	+0.2	+0.08	+10
9	68/F	Worsened	−1	−3.0	+0.2	+0.02	−3
Median			+1	−0.8	±0.0	+0.02	+10

CAT  =  chronic obstructive pulmonary disease assessment test; BW  =  body weight; ALB  =  albumin; CRP  =  C-reactive protein; ESR  =  erythrocyte segmentation rate.

*Chronic obstructive pulmonary disease assessment test score ranging from 0 to 40. A higher score means more severe symptoms [11].

### Safety

None of the patients experienced severe adverse events greater than grade 3. In the Hochuekkito group, two patients developed adverse events. One patient developed pneumonitis (grade 3 on day 54), which was improved by discontinuing Hochuekkito and administering intravenous antibiotics (piperacillin/tazobactam). The possibility of Hochuekkito-induced pneumonitis was not excluded. The other patient experienced oral mucositis (grade 2), limb edema (grade 1), diarrhea (grade 1), and a maculopapular rash (grade 1) from day 3 to the end of follow up. Two patients in the control group also experienced adverse events. One developed a lung infection (grade 2 on day 53), which was treated by oral antibiotics (amoxicillin/clavulanate). The other patient developed an optic nerve disorder (grade 2 on day 72) that was induced by ethambutol.

## Discussion

Although the sample size was very small, to the best of our knowledge, this is the first prospective comparative study to evaluate the efficacy and safety of Hochuekkito in patients with progressed pulmonary MAC disease. None of the patients in either group achieved sputum conversion despite the 24-week continuation of the baseline treatment with or without Hochuekkito. Nevertheless, we did observe several clinically relevant results and these, in combination with our safety analysis, suggesting that Hochuekkito may be feasible for use because it appears to have several beneficial effects.

It is surprising that 8/9 patients in the Hochuekkito group were able to control the disease progression (assessed by the radiological assessment), particularly because they had more severe status with significantly higher ESR than the control group. This result is partially consistent with the changes in the number of MAC colonies in sputum culture. Most patients in our cohort were elderly, had a low body mass index, and had cavities observed in chest X-ray, all of which have been reportedly poor prognostic factors in pulmonary MAC disease [12]. In addition, about half of the patients in each group had clarithromycin-resistant MAC, which is linked to the low sputum conversion rate [13]. Given the fact that the patients in this study had multiple disadvantages, and that their disease was poorly controlled by the preceding conventional treatment, it is noteworthy that some patients in the Hochuekkito group showed a partial response.

Previous studies have highlighted the immunomodulating effects of Hochuekkito. Some reports have suggested that administration of the drug increases serum interferon-γ levels, which is believed to be related to increased natural killer cell activity [6], [7], [14], [15]. Nakayama et al. reported the possibility of using Hochuekkito for recovering impaired tumor necrosis factor-α production in alveolar macrophages in hyperglycemic mice [16]. They suggested that Hochuekkito may normalize the dysregulated inflammatory response. Interferon-γ and tumor necrosis factor-α play important roles in controlling intracellular infections including MAC diseases [1], [17], [18]. These mechanisms may thus explain the effectiveness of this drug.

In the present study, interestingly, patients in the Hochuekkito group tended to maintain or gain body weights, and their serum albumin levels tended to remain stable or increased. Although these findings were not based on the statistical evaluation, these imply Hochuekkito may have a favorable impact on nutritional status. In a previous report on patients with moderate-to-severe chronic obstructive pulmonary disease, Hochuekkito was suggested to increase body weight and serum prealbumin levels [8], which is consistent with our findings. Another herbal drug, Rikkunshito, has been reported to improve anorexia by increasing ghrelin secretion [19], [20]. Six of the eight materials present in Rikkunshito (*Atractylodis rhizoma*, *Aurantii nobilis pericarpium*, *Gurantii radix*, *Glycyrrhizae radix*, *Zingiberis rhizoma*, and *Zizyphi fructus*) are common to Hochuekkito. Thus similar effectiveness might be expected. Management of nutritional status is a very important theme in chronic and wasting diseases, including progressed pulmonary MAC. Although it is unclear whether changes in nutritional status are associated with clinical outcome, in fact, all but one (6 of 7) patient who gained body weight in both groups of our study improved their X-ray images or remained stable. Our findings appear to suggest a new therapeutic target for this disease, and Hochuekkito may have particular strengths in this context.

There were several limitations to this study. First, a small number of patients were recruited, which did not provide the study with sufficient statistical power to prove efficacy. Our results should be regarded to be preliminary. Because of the strict criteria for patient entry, it was difficult to recruit additional patients. Second, in open-label study designs, there is a possibility that patients and clinicians may report biased answers, although the majority of our endpoints were considered to be relatively objective. Third, there were several small imbalances at baseline between the treatment groups particularly in terms of the smoking status, preceding treatment regimens, and disease severity. These differences may have influenced several outcomes. To confirm our preliminary findings, further placebo-controlled and large-scale studies are needed.

In conclusion, this pilot study suggests that Hochuekkito could be a feasible and beneficial adjunct to conventional treatment in patients with progressed pulmonary MAC disease, particularly among those for whom no alternative treatments are available. Our results should be confirmed by larger studies in future.

## Supporting Information

Protocol S1 **Trial protocol.**
(PDF)

Checklist S1 **CONSORT checklist.**
(DOC)
