# Supplementary material for: Pilot Quasi-Randomized Controlled Study of Herbal Medicine Hochuekkito as an Adjunct to Conventional Treatment for Progressed Pulmonary Mycobacterium avium Complex Disease
Source: PLoS One. 2014 Aug 5;9(8):e104411. doi: 10.1371/journal.pone.0104411 (PMC4122490; doi:10.1371/journal.pone.0104411)
Supplement: Protocol S1 — Trial protocol. (PDF) [file pone.0104411.s001.pdf]

UMIN CTR 臨床試験登録情報の閲覧

BACK | TOP ● UMIN-CTRホーム ● 用語の説明(簡易版) ● 用語の説明(詳細版)--準備中 ● FAQ

試験進捗状況 : **試験終了/Completed**
UMIN試験ID : UMIN000009920
試験名 : 治療抵抗性または不耐性肺MAC症における補中益気湯の有効性の検討(無作為化比較試験)
登録日(=情報公開日) : 2013/02/01
最終データ内容更新日時 : 2013/12/19 22:11:22

※ 本ページ収載の情報は、臨床試験に関する情報公開を目的として、UMINが開設しているUMIN臨床試験登録システムに提供された臨床試験情報です。
※ 特定の医薬品や治療法等については、医療関係者や一般の方に向けて広告することは目的としていません。

基本情報 (Basic information)		
項目(Item)	日本語(Japanese)	英語(English)
試験名 (Official scientific title of the study)	治療抵抗性または不耐性肺MAC症における補中益気湯の有効性の検討(無作為化比較試験)	Randomized controlled trial to evaluate anti-mycobacterial effects of Hochhu-ekkito add-on therapy for patients with pulmonary Mycobacterium avium complex disease intractable with or intolerant to conventional treatmnt
試験簡略名 (Title of the study (Brief title))	肺MAC症における補中益気湯の有効性の検討	Randomized controlled trial to evaluate anti-mycobacterial effects of Hochhu-ekkito add-on therapy for patients with pulmonary Mycobacterium avium complex disease
試験実施地域 (Region)	日本/Japan	

対象疾患(Condition)		
項目(Item)	日本語(Japanese)	英語(English)
対象疾患名 (Condition)	肺非結核性抗酸菌症(肺MAC症)	Pulmonary Mycobacterium avium complex disease
疾患区分1 (Classification by specialty)	呼吸器内科学/Pneumology	
疾患区分2 (Classification by malignancy)	悪性腫瘍以外/Others	
ゲノム情報の取扱い (Genomic information)	いいえ/NO	

目的(Objectives)		
項目(Item)	日本語(Japanese)	英語(English)
目的1 (Narrative	従来の治療を以ってしても喀痰培養陰転化が得られていない治療抵抗性肺MAC症あるいは、副作用等の問題で標準治療ができていない治療不耐性肺MAC症の患者を対象に、補	To evaluate anti-mycobacterial effects of Hochu-ekkito add-on therapy for patients with pulmonary Mycobacterium avium

objectives1)	中益気湯を追加することの有効性を検証する。	complex disease intractable with or intolerant to conventional treatment
目的2 (Basic objectives2)	安全性・有効性/Safety,Efficacy	
目的2 -その他詳細 (Basic objectives - Others)		
試験の性質1 (Trial characteristics_1)	探索的/Exploratory	
試験の性質2 (Trial characteristics_2)	実務的/Pragmatic	
試験のフェーズ (Developmental phase)	該当せず/Not applicable	

評価 (Assessment)		
項目(Item)	日本語(Japanese)	英語(English)
主要アウトカム評価項目 (Primary outcomes)	6ヶ月治療後の喀痰培養陰転化率	Sputum conversion rate over the six months treatment period
副次アウトカム評価項目 (Key secondary outcomes)	(1) 胸部レントゲン写真の変化<br>(2) QOL、症状の変化<br>(3) 体重の変化<br>(4) バイオマーカーの変化<br>(5) 有害事象の解析	(1) Changes in images of chest X-ray<br>(2) Changes in the scores for quality of life and symptoms<br>(3) Changes in body weight<br>(4) Changes in biomarkers measured during treatment<br>(5) Evaluation of adverse events

基本事項 (Base)		
項目(Item)	日本語(Japanese)	英語(English)
試験の種類 (Study type)	介入/Interventional	

試験デザイン (Study design)		
項目(Item)	日本語(Japanese)	英語(English)
基本デザイン (Basic design)	並行群間比較/Parallel	
ランダム化 (Randomization)	ランダム化/Randomized	
ランダム化の単位 (Randomization unit)	個別/Individual	
ブラインド化 (Blinding)	オープン/Open -no one is blinded	
コントロール (Control)	実薬・標準治療対照/Active	
層別化 (Stratification)	はい/YES	

動的割付<br>(Dynamic allocation)	
試験実施施設の考慮<br>(Institution consideration)	
ブロック化<br>(Blocking)	
割付コードを知る方法<br>(Concealment)	

介入 (Intervention)		
項目(Item)	日本語(Japanese)	英語(English)
群数<br>(No. of arms)	2	
介入の目的<br>(Purpose of intervention)	治療・ケア/Treatment	
介入の種類<br>(Type of intervention)	医薬品/Medicine	
介入1<br>(Interventions/Control_1)	介入群:<br>登録前から行われていた治療内容(登録時無治療であれば経過観察)に加え、補中益気湯5.0～7.5gを試験期間中毎日内服する	Treatment group:<br>Oral daily administration of Hochu-ekkito (5.0-7.5 g/day) plus the treatment currently performed at the time of registration. If the patients are not on any anti-mycobacterial chemotherapy, they will receive Hochu-ekkito alone.
介入2<br>(Interventions/Control_2)	対照群:<br>登録前から行われていた治療内容(登録時無治療であれば経過観察)をそのまま継続する	Control group:<br>Continuation of the treatment currently performed at the time of registration. If the patients are not on any anti-mycobacterial chemotherapy, they will be observed without medication.
介入3<br>(Interventions/Control_3)		
介入4<br>(Interventions/Control_4)		
介入5<br>(Interventions/Control_5)		
介入6<br>(Interventions/Control_6)		
介入7<br>(Interventions/Control_7)		
介入8<br>(Interventions/Control_8)		
介入9<br>(Interventions/Control_9)		
介入10<br>(Interventions/Control_10)		

適格性 (Eligibility)		
項目(Item)	日本語(Japanese)	英語(English)

年齢(下限)<br>(Age-lower limit)	20 歳/years-old 以上/<=	
年齢(上限)<br>(Age-upper limit)	適用なし/Not applicable	
性別<br>(Gender)	男女両方/Male and Female	
選択基準<br>(Key inclusion criteria)	(1) 同意取得時20歳以上である<br>(2) 登録より少なくとも1年以上前に肺MAC症と診断されている<br>(3) 登録3ケ月以内の喀痰培養検査でMACが検出されている<br>(4) 従来の治療が1年以上継続して行われている治療抵抗例、あるいは副作用等の問題で治療ができていない治療不耐例<br>(5)「比較的体力が低下し、全身倦怠感や食欲不振等を訴える場合」という補中益気湯使用目標(ツムラ補中益気湯エキス顆粒の医薬品インタビューフォーム参照)を満たす	(1) Patients over 20 years old<br>(2) Patients who has been already diagnosed as having pulmonary mycobacterium avium complex disease at least one year ago<br>(3) Patients confirmed with positive sputum culture for mycobacterium avium complex within three months<br>(4) Patients who has been receiving conventional therapy for one year or more but intractable, or patients who had undergone conventional chemotherapy but become intolerant to it<br>(5) Patients who fulfill the recommended conditions for use of Hochu-ekkito: relatively declined physical strength, suffering from general fatigue and/or appetite loss.
除外基準<br>(Key exclusion criteria)	(1) 悪性疾患治療中である<br>(2) 間質性肺疾患を有している<br>(3) 登録3ケ月以内に抗MAC治療薬の種類の変更をしている<br>(4) 登録3ケ月以内に補中益気湯を含む医療用漢方薬を使用している<br>(5) 他にコントロール不良の呼吸器疾患を有している<br>(6) 妊娠中あるいは妊娠を予定している<br>(7) その他医師が不適当と判断した場合	(1) Patients with any malignant diseases<br>(2) Patients with interstitial lung diseases<br>(3) Patients whose regimen for anti-mycobacterai therapy had been changed within three months<br>(4) Patients who had been receiving any herbal drugs including Hochu-ekkito within three months<br>(5) Patients with severe other pulmonary diseases<br>(6) Pregnant women or females with childbearing potential<br>(7) Any other condition which, in the opinion of the investigator, would make the subject unsuitable for enrollment
目標参加者数<br>(Target sample size)	80	

責任研究者 (Research contact person)

項目(Item)	日本語(Japanese)	英語(English)
責任研究者名<br>(Name of lead principal investigator)	榎本　泰典	Yasunori Enomoto
所属組織<br>(Organization)	神奈川県立循環器呼吸器病センター	Kanagawa Cardiovascular and Respiratory Center
所属部署<br>(Division name)	呼吸器内科	Division of Respiratory Medicine
住所<br>(Address)	〒236-0051 神奈川県横浜市金沢区富岡東6-16-1	6-16-1 Tomioka-Higashi, Kanazawa-ku, Yokohama City 236-0051, Japan
電話<br>(TEL)	045-701-9581	
Email<br>(Email)	yasuyasuyasu29@yahoo.co.jp	

試験問い合わせ窓口(Public contact)		
項目(Item)	日本語(Japanese)	英語(English)
担当者名 (Name of contact person)	榎本　泰典	Yasunori Enomoto
組織名 (Organization)	神奈川県立循環器呼吸器病センター	Kanagawa Cardiovascular and Respiratory Center
部署名 (Division name)	呼吸器内科	Division of Respiratory Medicine
住所 (Address)	神奈川県横浜市金沢区富岡東6-16-1	6-16-1 Tomioka-Higashi, Kanazawa-ku, Yokohama City 236-0051, Japan
電話 (TEL)	045-701-9581	
試験のホームページURL (Homepage URL)		
Email (Email)	yasuyasuyasu29@yahoo.co.jp	

実施責任組織 (Sponsor)		
項目(Item)	日本語(Japanese)	英語(English)
実施責任組織 (Name of primary sponsor)	神奈川県立循環器呼吸器病センター	Kanagawa Cardiovascular and Respiratory Center

実施責任組織とは、「試験の計画、解析と結果公表、研究費調達を含めた実施のための運営管理に対して責任を持つ組織」です。英語名でスポンサーとありますが、通常イメージする資金提供者のことではございません。従いまして、「なし」という記載はありえません。

研究費提供組織(Funding Source)		
項目(Item)	日本語(Japanese)	英語(English)
研究費提供組織 (Source of funding)	なし	None
組織の区分 (Category of Org.)	自己調達/Self funding	
研究費拠出国 (Nation of funding)		

その他の関連組織 (Other related organizations)		
項目(Item)	日本語(Japanese)	英語(English)
共同実施組織 (Name of secondary sponsor(s))		
その他の研究費提供組織 (Name of secondary funder(s))		

他機関から発行された試験ID (Secondary study IDs)		
項目(Item)	日本語(Japanese)	英語(English)
他機関から発行された試験ID (Secondary study IDs)	いいえ/NO	
試験ID1 (Secondary study ID_1)		
ID発行機関1 (Org. issuing Secondary study ID_1)		
試験ID2 (Secondary study ID_2)		
ID発行機関2 (Org. issuing Secondary study ID_2)		
治験届 (IND to MHLW)		

試験実施施設 (Institutions)		
項目(Item)	日本語(Japanese)	英語(English)
試験実施施設名称 (Institutions)	神奈川県立循環器呼吸器病センター	

試験進捗状況 (Progress)		
項目(Item)	日本語(Japanese)	英語(English)
試験進捗状況 (Recruitment status)	試験終了/Completed	
プロトコル確定日 (Date of protocol fixation)	2013/01/31	
登録・組入れ開始(予定)日 (Anticipated trial start date)	2013/02/01	
フォロー終了(予定)日 (Last follow-up date)	2014/02/28	
入力終了(予定)日 (Date of closure to data entry)		
データ固定(予定)日 (Date trial data considered complete)	2013/12/19	
解析終了(予定)日 (Date analysis concluded)	2013/12/19	

関連情報 (Related information)		
項目(Item)	日本語(Japanese)	英語(English)
プロトコル掲載URL (URL releasing protocol)		
試験結果の公開状況 (Publication of results)	未公表/Unpublished	
結果掲載URL (URL releasing results)		
主な結果 (Results)		
その他関連情報 (Other related information)		

管理情報		
項目(Item)	日本語(Japanese)	英語(English)
登録日 (Date of registration)	2013/02/01	
最終情報更新日 (Date of last update)	2013/12/19 22:11:22	

閲覧ページへのリンク	
日本語URL	https://upload.umin.ac.jp/cgi-open-bin/ctr/ctr.cgi?function=brows&action=brows&recptno=R000011622&type=summary&language=J
英語URL	https://upload.umin.ac.jp/cgi-open-bin/ctr/ctr.cgi?function=brows&action=brows&recptno=R000011622&type=summary&language=E

※ 本ページ収載の情報は、臨床試験に関する情報公開を目的として、UMINが開設しているUMIN臨床試験登録システムに提供された臨床試験情報です。

※ 特定の医薬品や治療法等については、医療関係者や一般の方に向けて広告することは目的としていません。

戻る

UMIN臨床試験登録システムのご使用に関するお問い合わせは、こちらのお問い合わせフォーム からお願いいたします。それ以外のお問い合わせは、こちら よりお願い致します。

UMIN Infrastructure for Academic Activities
University hospital Medical Information Network

UMIN-CTR Clinical Trial

BACK | TOP

**Recruitment status**	:	**Completed**
Unique trial Number	:	UMIN000009920
Title of the study	:	Randomized controlled trial to evaluate anti-mycobacterial effects of Hochhu-ekkito add-on therapy for patients with pulmonary Mycobacterium avium complex disease intractable with or intolerant to conventional treatmnt
Date of formal registration (=Date of ICMJE and WHO compliant trial information registration and disclosure)	:	2013/02/01
Date and time of last update	:	2013/12/19 22:11:22

※ This page includes information on clinical trials registered in UMIN clinical trial registed system.

※ We don't aim to advertise certain products or treatments.

Basic information	
Item	Value
Official scientific title of the study	Randomized controlled trial to evaluate anti-mycobacterial effects of Hochhu-ekkito add-on therapy for patients with pulmonary Mycobacterium avium complex disease intractable with or intolerant to conventional treatmnt
Title of the study (Brief title)	Randomized controlled trial to evaluate anti-mycobacterial effects of Hochhu-ekkito add-on therapy for patients with pulmonary Mycobacterium avium complex disease
Region	Japan

Condition	
Item	Value
Condition	Pulmonary Mycobacterium avium complex disease
Classification by specialty	Pneumology
Classification by malignancy	Others
Genomic information	NO

Objectives	
Item	Value
Narrative objectives1	To evaluate anti-mycobacterial effects of Hochu-ekkito add-on therapy for patients with pulmonary Mycobacterium avium complex disease intractable with or intolerant to conventional treatment
Basic objectives2	Safety,Efficacy
Basic objectives -Others	
Trial characteristics_1	Exploratory

Trial characteristics_2	Pragmatic
Developmental phase	Not applicable

Assessment	
Item	Value
Primary outcomes	Sputum conversion rate over the six months treatment period
Key secondary outcomes	(1) Changes in images of chest X-ray<br>(2) Changes in the scores for quality of life and symptoms<br>(3) Changes in body weight<br>(4) Changes in biomarkers measured during treatment<br>(5) Evaluation of adverse events

Base	
Item	Value
Study type	Interventional

Study design	
Item	Value
Basic design	Parallel
Randomization	Randomized
Randomization unit	Individual
Blinding	Open -no one is blinded
Control	Active
Stratification	YES
Dynamic allocation	
Institution consideration	
Blocking	
Concealment	

Intervention	
Item	Value
No. of arms	2
Purpose of intervention	Treatment
Type of intervention	Medicine
Interventions/Control_1	Treatment group:<br>Oral daily administration of Hochu-ekkito (5.0-7.5 g/day) plus the treatment currently performed at the time of registration. If the patients are not on any anti-mycobacterial chemotherapy, they will receive Hochu-ekkito alone.
Interventions/Control_2	Control group:<br>Continuation of the treatment currently performed at the time of registration. If the patients are not on any anti-mycobacterial chemotherapy, they will be observed without medication.
Interventions/Control_3	

Interventions/Control_4	
Interventions/Control_5	
Interventions/Control_6	
Interventions/Control_7	
Interventions/Control_8	
Interventions/Control_9	
Interventions/Control_10	

Eligibility

Item	Value
Age-lower limit	20 years-old <=
Age-upper limit	Not applicable
Gender	Male and Female
Key inclusion criteria	(1) Patients over 20 years old<br>(2) Patients who has been already diagnosed as having pulmonary mycobacterium avium complex disease at least one year ago<br>(3) Patients confirmed with positive sputum culture for mycobacterium avium complex within three months<br>(4) Patients who has been receiving conventional therapy for one year or more but intractable, or patients who had undergone conventional chemotherapy but become intolerant to it<br>(5) Patients who fulfill the recommended conditions for use of Hochu-ekkito: relatively declined physical strength, suffering from general fatigue and/or appetite loss.
Key exclusion criteria	(1) Patients with any malignant diseases<br>(2) Patients with interstitial lung diseases<br>(3) Patients whose regimen for anti-mycobacterai therapy had been changed within three months<br>(4) Patients who had been receiving any herbal drugs including Hochu-ekkito within three months<br>(5) Patients with severe other pulmonary diseases<br>(6) Pregnant women or females with childbearing potential<br>(7) Any other condition which, in the opinion of the investigator, would make the subject unsuitable for enrollment
Target sample size	80

Research contact person

Item	Value
Name of lead principal investigator	Yasunori Enomoto
Organization	Kanagawa Cardiovascular and Respiratory Center
Division name	Division of Respiratory Medicine
Address	6-16-1 Tomioka-Higashi, Kanazawa-ku, Yokohama City 236-0051, Japan
TEL	+81-45-701-9581
Email	yasuyasuyasu29@yahoo.co.jp

Public contact

Item	Value
Name of contact person	Yasunori Enomoto
Organization	Kanagawa Cardiovascular and Respiratory Center
Division name	Division of Respiratory Medicine
Address	6-16-1 Tomioka-Higashi, Kanazawa-ku, Yokohama City 236-0051, Japan
TEL	+81-45-701-9581
Homepage URL	
Email	yasuyasuyasu29@yahoo.co.jp

Sponsor	
Item	Value
Name of primary sponsor	Kanagawa Cardiovascular and Respiratory Center

Sponsor means an organization that is responsible for plan, deployment and report of the research including funding management. It doesn't mean "funding agency". Therefore, all clinical trial should have the one.

Funding Source	
Item	Value
Source of funding	None
Category of Org.	Self funding
Nation of funding	

Other related organizations	
Item	Value
Name of secondary sponsor(s)	
Name of secondary funder(s)	

Secondary study IDs	
Item	Value
Secondary study IDs	NO
Secondary study ID_1	
Org. issuing Secondary study ID_1	
Secondary study ID_2	
Org. issuing Secondary study ID_2	
IND to MHLW	

Institutions	
Item	Value
Institutions	神奈川県立循環器呼吸器病センター

Progress	
Item	Value
Recruitment status	Completed
Date of protocol fixation	2013/01/31
Anticipated trial start date	2013/02/01
Last follow-up date	2014/02/28
Date of closure to data entry	
Date trial data considered complete	2013/12/19
Date analysis concluded	2013/12/19

Related information	
Item	Value
URL releasing protocol	
Publication of results	Unpublished
URL releasing results	
Results	
Other related information	

Item	Value
Date of registration	2013/02/01
Date of last update	2013/12/19 22:11:22

Link to view	
URL(Japanese)	https://upload.umin.ac.jp/cgi-open-bin/ctr/ctr.cgi?function=brows&action=brows&recptno=R000011622&type=summary&language=J
URL(English)	https://upload.umin.ac.jp/cgi-open-bin/ctr/ctr.cgi?function=brows&action=brows&recptno=R000011622&type=summary&language=E

※ This page includes information on clinical trials registered in UMIN clinical trial registed system.

※ We don't aim to advertise certain products or treatments.

Back

UMIN Infrastructure for Academic Activities
University hospital Medical Information Network
